# Categorical Risk Stratification for Neonatal Early Onset Sepsis using Suspected Intraamniotic Infection and the Newborn Exam

**DOI:** 10.21203/rs.3.rs-2838294/v1

**Published:** 2023-08-01

**Authors:** Anne-Marie Rick, Elizabeth Copp, Abigail Buckley, Toby Yanowitz, Judith Martin, Nader Shaikh, Galen Switzer, Thomas Hooven, Richard Beigi

**Affiliations:** University of Pittsburgh; University of Pittsburgh; University of Pittsburgh; Three Rivers Rowing Association; University of Pittsburgh; University of Pittsburgh; University of Pittsburgh; University of Pittsburgh; University of Pittsburgh

## Abstract

**Objective:**

To determine test characteristics of categorical risk stratification for early onset sepsis (EOS) using maternal criteria for suspected intraamniotic infection (IAI) and/or newborn exam and compare them to the EOS calculator.

**Study Design:**

Retrospective 1:3 case-control study of late preterm/term infants with bacterial culture growth obtained <72 hours of life. For categorical approach, infants of mothers with suspected IAI or equivocal/ill appearing were presumed high-risk for EOS and blood culture obtained. For calculator, estimated probability of EOS and care recommendations were recorded from online calculator. Test characteristics were compared with McNemar’s test; recommendation for blood culture was considered a “positive” test.

**Result:**

52 cases and 172 controls were included. Compared to the calculator, the categorical approach had higher sensitivity 90%(95%CI:79–96%) vs 67% (95%CI:54–79%) but lower specificity 85%(95%CI:78–89%) vs. 92%(95%CI:87–96%). 10% of cases were not identified by either.

**Conclusion:**

A categorical approach using suspected IAI/newborn exam offers good EOS discrimination and is comparable to the calculator.

## Introduction

In 2018, the American Academy of Pediatrics (AAP) revised national guidelines to recommend several approaches to identify infants ≥ 35 weeks gestational age (GA) at increased risk of early onset sepsis (EOS). Two of these approaches include: 1) categorical risk factor assessment and 2) multivariate risk assessment.(1) Each approach aims to identify infants with EOS to allow appropriate intervention and treatment while minimizing the identification of uninfected infants to avoid unnecessary antibiotic exposure, laboratory evaluations and disruption of maternal/infant bonding.

For the categorical approach, the AAP guidelines suggest using the obstetric diagnosis of clinical chorioamnionitis or a maternal intrapartum fever ≥ 38°C as a threshold for neonatal EOS screening.(2, 3) Indeed, over 60% of nurseries identify using this as their approach to EOS risk stratification.(4) However, as of 2017, the American College of Obstetrics and Gynecology (ACOG) recommends transitioning away from the terminology and historical criteria for clinical chorioamnionitis and instead recommends new clinical criteria (see methods), which are used to diagnosis women with suspected intraamniotic infection.(3, 5) If present, this condition warrants intrapartum antibiotic therapy.(5) Although obstetricians across the country now apply these new diagnostic criteria to women in delivery, limited studies have evaluated how a maternal diagnosis of suspected intraamniotic infection relates to the identification of EOS in infants. (6) This is problematic because despite being diagnostically distinct from clinical chorioamnionitis, in clinical practice the diagnosis of suspected intraamniotic infection is often conflated with the diagnosis of clinical chorioamnionitis for purposes of EOS risk stratification. Therefore, many well-baby units may unknowingly shift who they identify as high-risk for EOS due to changes in obstetric practice. Until it is evaluated, the impact of this clinical change on EOS risk stratification is unknown.

Thus, there is a need to understand the diagnostic characteristics of this new maternal clinical diagnosis for EOS to inform clinicians as they evaluate which EOS risk stratification approach is most appropriate for their setting. Therefore, using a retrospective case-control design, we sought to evaluate the sensitivity, specificity, and area under the curve for a categorical approach using suspected intraamniotic infection and the infant clinical appearance for culture-confirmed EOS among infants ≥35 weeks GA. We further sought to contextualize these findings by providing a side-to-side comparison of test characteristics to a multivariate risk assessment approach using the highly evidenced-based Neonatal EOS Risk Calculator (hereon referred to as calculator).(7–9) This web-based calculator incorporates an infant’s individual risk factors and clinical appearance to identify a continuous probability of EOS, which is then grouped into five levels of risk and suggestions for clinical management are provided ranging from enhanced observation to neonatal intensive care unit (NICU) transfer, blood culture and antibiotics. We hypothesized that the categorical approach using criteria for suspected intraamniotic infection would improve discrimination between infants with and without culture-confirmed EOS compared to historical approaches using criteria for clinical chorioamnionitis. However, we hypothesized that the multivariate approach would demonstrate overall better discrimination.

## Methods

This was a single-institution, retrospective nested case-control study of mother-infant pairs who delivered at ≥ 35 weeks GA from June 1, 2008 to December 31, 2020. Using an electronic health record (EHR) query, we first identified infants with bacterial growth identified on blood (peripheral or cord) culture or cerebral spinal fluid (CSF) culture obtained within 72 hours of life using an automated continuous detection culture system. We then excluded infants who were < 35 weeks GA, who had positive cultures and were treated with less than five days of antibiotics or cultures with likely bacterial contaminants (i.e. coagulase negative staph species)(10), who were born outside of the hospital, readmitted or with significant anomalies as defined by the Vermont-Oxford Neonatal Network (www.vtoxford.org). We also excluded infants whose mothers had no recorded temperatures prior to delivery or no documented time of membrane rupture. Remaining infants were included as cases of culture-confirmed EOS. Using a statistical program, we then randomly selected three control infants ≥ 35 weeks GA and born within a year of each case through the Magee Obstetric Medical and Infant (MOMI) electronic database, which collects real-time data on > 95% of births at Magee Women’s Hospital from the EHR. We excluded controls who met any of the exclusion criteria described above, as well as those who received five or more days of antibiotics in the absence of a positive culture. Additional random controls were then selected to replace those excluded. Notably, at our institution for the years included all infants ≥ 35 weeks GA born to a mother with a diagnosis of clinical chorioamnionitis, which at the time would have been based on historical criteria, were empirically evaluated for EOS and treated with 48 hours of antibiotics pending culture results.

## Data Collection

We abstracted all demographic, prenatal and peripartum data necessary for the multivariate EOS calculator and categorical risk assessment including maternal and infant vital signs, laboratory data, and antibiotics administered from the EHR.(7) Duration of membrane rupture was calculated based on documented time of membrane rupture and time of delivery. Maternal intrapartum antibiotics were categorized as: none; antibiotic less than two hours prior to delivery; Group B Streptococcus (GBS) intrapartum antibiotic prophylaxis (IAP) (including penicillin, ampicillin, amoxicillin, clindamycin, cefazolin, vancomycin) at least two hours prior to delivery; broad-spectrum antibiotic (other cephalosporins, fluoroquinolone, or any antibiotic from IAP antibiotic plus aminoglycoside) between 2 to 3.99 hours prior to delivery; and broad-spectrum antibiotic at least 4 hours prior to delivery.(7–9) Fetal tachycardia was recorded if fetal heart rate > 160 beats per minute was recorded in labor vital sign flowsheets or in obstetric or neonatology notes. Purulence of amniotic fluid was identified exclusively from obstetric and neonatology notes. For infant clinical appearance, data on vital signs, Apgar score at 5 minutes of life as well as any oxygen or vasopressor support, and presence of seizures < 12 hours of life were recorded from flowsheets and notes.

## Risk Stratification for EOS

Categorical EOS Risk Assessment: Categorical risk was determined using criteria for suspected intraamniotic infection in the mother based on ACOG’s 2017 guidelines or if an infant developed equivocal or clinical illness during the first 12 hours of life. Suspected intraamniotic infection was diagnosed based on a maternal temperature of ≥ 39.0°C or maternal temperature of 38.0–38.9°C with either presence of maternal white blood cells > 15,000 cells/cubic millimeter (mm^3^), fetal heart rate > 160 beats per minute or amniotic purulence. Infant clinical appearance was categorized retrospectively as well-appearing, equivocal or clinical illness based on the criteria for vital sign patterns and clinical presentation defined by the multivariate EOS risk calculator occurring during the first 12 hours of life (**Supplemental Table 1**).(8, 11) (5). If the infant met criteria for equivocal or clinical illness at any point during the first 12 hours then this was used as their appearance. For purposes of this analysis, infants who were exposed to suspected intraamniotic infection and/or had equivocal or clinical illness presentation were considered “high-risk” and presumed to be managed with a blood culture and empiric antibiotics, while all other infants were considered “low-risk” and would receive routine care.

Neonatal EOS Risk Calculator: The calculator is available for public use at https://neonatalsepsiscalculator.kaiserpermanente.org and accessed from May to June 2023 for calculation for this study. To establish each infant’s probability of EOS at birth and to obtain the calculator’s management recommendations, we utilized our cohort’s incidence of EOS and entered peripartum factors including gestational age, highest maternal temperature in Celsius, duration of membrane rupture, GBS status, and antibiotic exposure for each infant, as well as the infant’s clinical appearance during the first 12 hours of life as defined above and in Supplemental Table 1.(8, 11) The calculator’s estimated probability of EOS and its clinical and vital sign monitoring recommendations were recorded. Clinical recommendations include no blood culture or antibiotics, blood culture only, strongly consider blood culture and empiric antibiotics, and blood culture and empiric antibiotic. Vital sign recommendations include routine vitals, vitals every 4 hours for 24 hours, or vitals per the NICU.

## Statistical Analysis

### Comparison of group characteristics

We compared demographic and birth characteristics between the baseline population and controls and cases and controls using chi-square, Fisher’s exact and Student t-tests.

#### Test characteristics of risk stratification approaches.

We examined sensitivity and specificity with Wilson’s 95% confidence intervals for the calculator and the categorical approaches for EOS using results from infant blood or CSF cultures as the gold standard. Bacterial growth on blood or CSF culture indicated EOS, while no growth on blood or CSF cultures or absence of a culture indicated no infection. This is a necessary but reasonable assumption as cultures and antibiotics are not routine in well-appearing, low-risk infants but untreated, infected infants would typically progress to clinical illness within 48–72 hours of birth, at which time a culture would be obtained.(1, 12) For both approaches, we considered a recommendation for obtaining a blood culture as a “positive” test.

We compared sensitivity and specificity for the two approaches using a McNemar test and the area under the receiver operating curve (AUC) using DeLong chi-squared test. Differences were considered statistically significant at a p-value of 0.05 or less. We then conducted three sensitivity analyses. First, for the categorical approach, we reclassified infants with equivocal clinical appearance as “low-risk” to assess impact on results. Second, for the calculator, we assessed test characteristics after adjusting our “positive” test definition to include the recommendation for vitals at least every four hours for 24 hours. Third, we excluded all positive blood cultures obtained from cord blood and reassessed test characteristics. For the calculator, we recalculated baseline EOS incidence in our cohort after cord blood cultures were excluded. All analyses were completed using Stata 15.0 (Stata Corp, College Station, Texas). This study was approved by the University of Pittsburgh institutional review board (PRO17110548).

## Results

**Figures 1:**
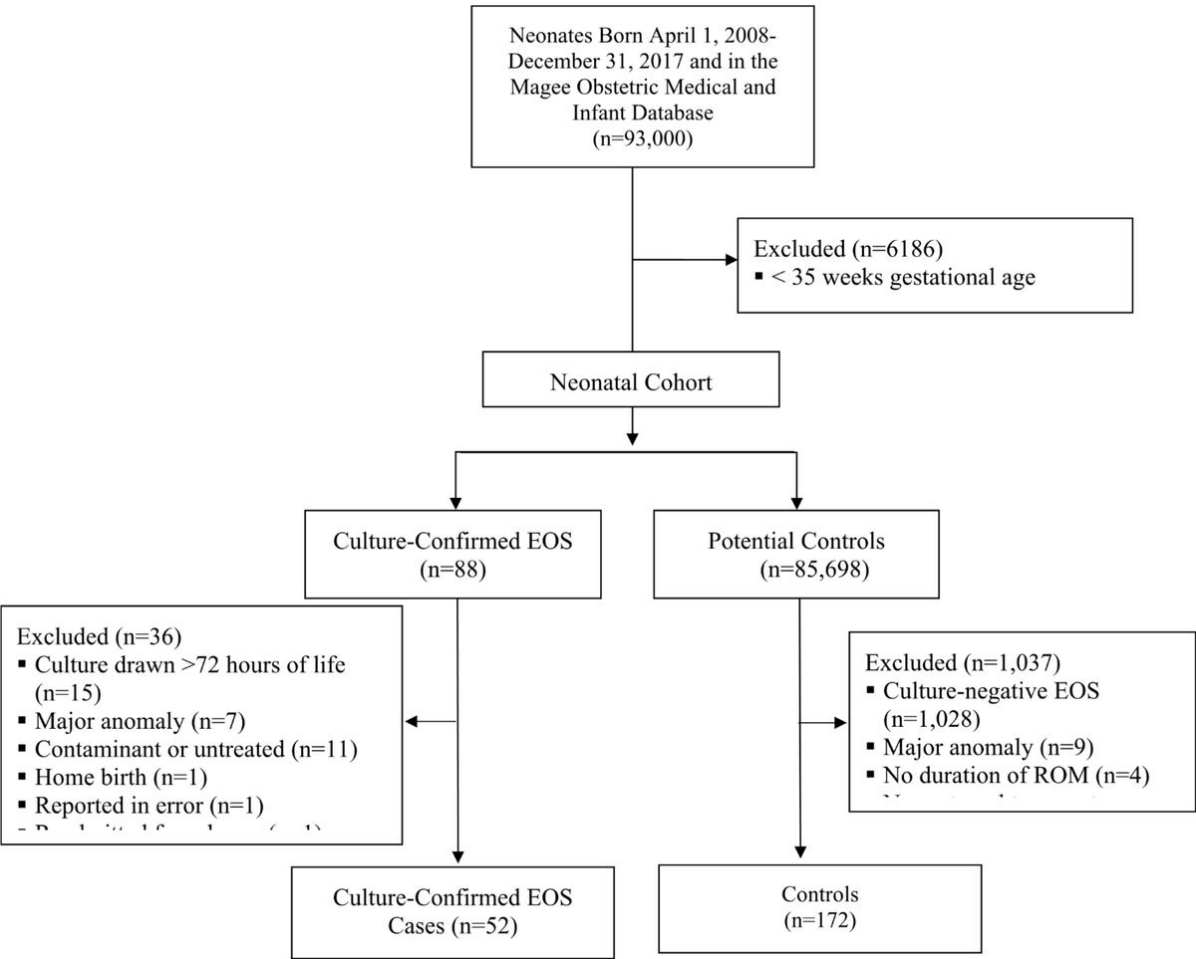
Flow diagram of included cases and controls.

Fifty-two cases and 156 controls were identified from 85,786 live-births ³ 35 weeks GA, giving a local EOS incidence of 0.60 cases per 1,000 live-births (Figure 1). Fifty (96%) cases had bacterial growth on blood cultures from peripheral (N=38) or cord blood specimens (N=12), and 2 (4%) cases had bacterial growth on cultures of CSF. GBS (34%, N=18) and *Escherichia coli* (19%, N=10) accounted for the majority of the infections. Demographic and peripartum factors for cases and controls are described in Table 1.

### Test Characteristics of the Calculator and Categorical Approaches

The categorical approach identified a total of 47 (90%) infants with culture-confirmed EOS and 24 (16%) controls. The calculator identified 35 (67%) infants with culture confirmed EOS and 12 (8%) controls (Table 2). Thus, the calculator had lower sensitivity for EOS (67%; 95%CI: 54–79%) compared to the categorical approach (90%; 95%CI:79–96%; p<0.001) (Table 3). Five (10%) cases of EOS were not identified by either approach (Table 4). Three of those infants developed clinical symptoms after 12 hours of life and two had mothers diagnosed with chorioamnionitis after delivery. Twelve (23%) cases were identified by the categorical approach only (Table 2). The calculator recommended vital signs every four hours for 24 hours for seven of these infants and routine vitals for five infants. A pattern of risk factors emerged among cases of EOS not identified by the calculator including well appearing infants ³ 37 weeks GA exposed to lower grade maternal fevers (38.0-C-39.1C) (N=11; Table 4).

Specificity was higher for the calculator (92%; 95%CI:87–96%) compared to the categorical approach (85%; 95%CI:78–89%; p=0.003) (Table 3). The two approaches were 90% concordant in ruling out controls. The AUC for the categorical approach (0.875; 95%CI:0.825–0.924) was significantly higher than AUC for the calculator (0.798 (95%CI: 0.730–0.865; p=0.016). Based on the NNTb, for every 34 infants evaluated with blood culture through the categorical approach as defined here, one infant with EOS would be identified. In contrast, for every 71 infants evaluated with blood culture through the calculator approach, one infant with EOS would be identified.

On sensitivity analysis, where we considered infants with an equivocal examination as low-risk for the categorical approach, the test characteristics of the categorical approach became quite similar to those of the calculator (Table 3). On the other hand, when we considered the recommendation to obtain vitals signs every four hours for 24 hours as a positive test for the calculator, we found the test characteristics for this approach became similar to those of the categorical approach (Table 3).

## Discussion

The results of our study suggest that a categorical approach using the combination of maternal suspected intraamniotic infection and infant clinical appearance has overall good test characteristics for the identification of EOS and would likely reduce overtreatment of uninfected infants compared to categorical approaches using the historical definition of clinical chorioamnionitis. This approach is overall comparable to the calculator for EOS risk stratification; however, its decreased specificity and empiric treatment approach means that more uninfected infants would still receive antibiotics compared to the calculator. Nevertheless, each approach has limitations that will result in some missed cases of EOS. Therefore, it is critical that vigilance through vital sign and physical examination monitoring is utilized for all infants regardless of maternal or peripartum risk factors.

## Conclusion

The results of our study suggest that a categorical approach using the combination of maternal suspected intraamniotic infection and infant clinical appearance has overall good test characteristics for the identification of EOS and would likely reduce overtreatment of uninfected infants compared to categorical approaches using the historical definition of clinical chorioamnionitis. This approach is overall comparable to the calculator for EOS risk stratification; however, its decreased specificity and empiric treatment approach means that more uninfected infants would still receive antibiotics compared to the calculator. Nevertheless, each approach has limitations that will result in some missed cases of EOS. Therefore, it is critical that vigilance through vital sign and physical examination monitoring is utilized for all infants regardless of maternal or peripartum risk factors.

## Figures and Tables

**Table 1.** Demographics and birth characteristics of cases and controls.

Characteristic	Cases (n=52)	Controls (n=156)

**Maternal Characteristics**		

Maternal Age, years, mean (SD)	28.5 (±4.9)	29.3 (±5.3)

Maternal Race		
White	30 (58)	106 (68)
Black	16 (31)	41 (26)
Other	6 (11)	9 (6)

Parity		
0	40 (77)	60 (38)
^3^ 1	12 (23)	96 (62)

Anesthesia		
Epidural	43 (82)	112 (72)
Other	7 (14)	27 (17)
None	2 (4)	17 (11)

Mode of Delivery		
Vaginal	31 (60)	111 (71)
Caesarian	21 (40)	45 (29)

**Infant Characteristics**		

Infant Sex		
Female	28 (54)	68 (44)
Male	24 (46)	88 (56)

Birth Weight		
< 2500 grams	3 (6)	6 (4)
^3^ 2500 grams	49 (94)	150 (96)

**Screening Tool Characteristics**		

Highest Maternal Temperature		
<38.0°C	21 (41)	150 (96)
38.0–38.9°C	22 (42)	6 (4)
^3^ 39.0°C	9 (17)	0 (0)

Maternal GBS status		
Negative	41 (79)	119 (76)
Positive	11 (21)	32 (21)
Unknown	0 (0)	5 (3)

Maternal Intrapartum Antibiotics		
None or < 2 hours prior to birth	38 (73)	121 (78)
GBS IAP ^3^ 2 hours prior to birth	8 (15)	31 (20)
Broad spectrum 2–3.9 hours prior to birth	1 (2)	0 (0)
Broad spectrum ^3^ 4 hours prior to birth	5 (10)	4 (2)

Rupture of Membranes		
<12 hours	20 (38)	127 (82)
12 to < 18 hours	13 (25)	14 (9)
18 to < 24 hours	6 (12)	9 (6)
^3^ 24 hours	13 (25)	4 (3)

Gestational Age, weeks		
35–36	6 (12)	13 (8)
37–38	9 (17)	30 (19)
39–40	30 (58)	96 (62)
≥41	7 (13)	17 (11)

Maternal white blood cell count		
<15,000 cells/mm^3^	26 (54)	101 (66)
^3^15,000 cells/mm^3^	17 (35)	11 (7)
Unknown	5 (10)	42 (27)

Fetal Tachycardia (^3^ 160 beats per minute)		
No	22 (42)	152 (97)
Yes	30 (58)	4 (3)

Mother meets Suspected Intraamniotic Infection criteria		
No		
Yes	23 (44)	152 (97)
	29 (56)	4 (3)

Infant Appearance 0–12 hours of birth		
Well-Appearing	28 (54)	136 (87)
Equivocal	9 (17)	14 (9)
Clinical Illness	15 (29)	6 (4)

SD: standard deviation; °C: Celsius; GBS: group B *Streptococcus;* IAP: Intrapartum antibiotic prophylaxis. Data are N (%) unless otherwise specified.

**Table 2.** Proportion of cases and controls identified by the categorical risk assessment and the multivariate EOS calculator approaches.

Categorical Risk Assessment				

	Cases	No blood culture	Blood culture	Total cases

**Multivariate EOS Calculator**	No blood culture	5 (10)	12 (23)	17 (33)
	Blood culture	0 (0)	35 (67)	33 (67)

	**Total cases**	5 (10)	47 (90)	52 (100)

	**Controls**	No blood culture	Blood culture	**Total controls**

	No blood culture	130 (83)	14 (9)	144 (92)
	Blood culture	2 (1)	10 (7)	12 (8)

	**Total controls**	132 (84)	24 (16)	156 (100)

EOS: early onset sepsis; Data are N (%).

**Table 3.** Sensitivity, specificity, AUC, and NNTb for culture-confirmed EOS by the categorical approach and multivariate EOS risk calculator.

Definition	Sensitivity % (95%CI)	Specificity % (95%CI)	AUC N (95%CI)	NNTb N (95%CI)

**Primary Analysis**				

Categorical	90 (79–96)	85 (78–89)	0.875 (0.825–0.924)	34 (13–95)

Multivariate EOS Calculator	67 (54–79)	92 (87–96)	0.798 (0.730–0.865)	71 (31–171)

**Sensitivity Analysis**				

Categorical: Exclude Equivocal	82 (70–91)	94 (89–97)	0.881 (0.727–0.863)	25 (10–66)
Examinations as Positives				

Multivariate EOS Calculator:	87 (75–93)	90 (84–94)	0.881 (0.805–0.919)	31 (13–81)
Vitals at Least Every 4 Hours is Positive				

AUC: area under the receiver operating curve; NNTb: number needed to test to benefit; EOS: early onset sepsis; 95%CI: 95% confidence interval.

**Table 4.** Characteristics of infants with culture-confirmed EOS identified neither or only by the categorical approach.

ID	GA (w, d)	Max. Maternal Temp. (°C)	ROM (hrs)	GBS Status	Maternal Antibiotics	Infant Exam	Infant Culture Pathogen*	Probability of EOS	Calculator Recommendation
**Identified by categorical approach only**									
1	37, 2	38.3	7.6	Neg	Broad >4hrs	Well	*L. monocytogenes*	0.22	Routine VS
2	39, 1	38.2	9.2	Pos	GBS IAP	Well	*A. neurii (cord)*	0.27	Routine VS
3	40, 6	38.1	3.3	Neg	None	Well	*S. gallolyticus*	0.30	Routine VS
4	39, 5	38.3	14.4	Pos	GBS IAP	Well	*S. mitis (cord)*	0.37	Routine VS
5	39, 0	38.1	14.3	Neg	None	Well	*S. mitis*	0.44	VS Q4hrs ×24h
6	39, 1	38.4	15.6	Pos	GBS IAP	Well	*E. coli*	0.45	VS Q4hrs ×24h
7	40, 2	38.1	17.1	Neg	None	Well	*E. faecalis*	0.48	VS Q4hrs ×24h
8	39, 6	38.0	26.7	Pos	None	Well	*S. agalactiae*	0.49	VS Q4hrs ×24h
9	41, 5	38.7	21.5	Neg	Broad >4hrs	Well	*S. agalactiae (cord)*	0.61	VS Q4hrs ×24h
10	39, 6	38.1	38.0	Neg	None	Well	*S. viridans*	0.68	VS Q4hrs ×24h
11	39, 1	39.1	24.0	Neg	Broad >4hrs	Well	*S. anginosus (cord)*	0.80	VS Q4hrs ×24h
12	35, 3	36.6	0.0	Pos	None	Equi.	*S. viridans*	0.98	Routine VS
**Not identified by either approach**									
1	38, 3	37.4	11.9	Pos	GBS IAP	Well	*S. aureus (csf)*	0.09	Routine VS
2	41, 5	37.9	16.5	Neg	None	Well	*S. aureus*	0.51	VS Q4hrs ×24h
3	40, 6	37.2	0.8	Neg	None	Well	*S. gallolyticus*	0.05	Routine VS
4	40, 1	38.4	24.2	Neg	None	Well	*E. coli*	0.89	VS Q4hrs ×24h
5	39, 5	37.8	5.5	Neg	None	Well	*E. coli*	0.19	Routine VS

EOS: early onset sepsis; GA: gestational age; w: weeks; d: days; Max: maximum; Temp: temperature; ROM: rupture of membranes; hrs: hours; GBS:

Group B Streptococcus; Neg: Negative; Pos: Positive; Broad: broad spectrum antibiotics administered more than 4 hours before delivery; IAP:

intrapartum antibiotic prophylaxis; Equi: equivocal; csf: cerebral spinal fluid; VS: vital signs; Q4hrs × 24 hrs: every 4 hours for 24 hours.

* Peripheral blood culture unless otherwise indicated in parentheses as cord blood or csf culture.

## Data Availability

Deidentified data are available upon request. Our institution is in the process of developing a repository for research datasets, where this data will be made available for public use.
